# Utility of Abdominal Drain in Gastrectomy (ADiGe) Trial: study protocol for a multicenter non-inferiority randomized trial

**DOI:** 10.1186/s13063-021-05102-1

**Published:** 2021-02-17

**Authors:** J. Weindelmayer, V. Mengardo, A. Veltri, G. L. Baiocchi, S. Giacopuzzi, G. Verlato, G. de Manzoni, on behalf of the Italian Research Group for Gastric Cancer (GIRCG)

**Affiliations:** 1 General and Upper G.I. Surgery Division, Azienda Ospedaliera Universitaria Integrata, Piazzale Aristide Stefani 1, Borgo Trento, 37126 Verona, Italy; 2 Department of Clinical and Experimental Sciences, University of Brescia, Brescia, Italy; 3 Department of Diagnostics and Public Health, University of Verona, Verona, Italy

**Keywords:** Gastric cancer, Gastrectomy, Abdominal drain, Drainage, Randomized controlled trial

## Abstract

**Background:**

Prophylactic use of abdominal drain in gastrectomy has been questioned in the last 15 years, and a 2015 Cochrane meta-analysis on four RCTs concluded that there was no convincing evidence to the routine drain placement in gastrectomy. Nevertheless, the authors evidenced the moderate/low quality of the included studies and highlighted how 3 out of 4 came from Eastern countries. After 2015, only retrospective studies have been published, all with inconsistent results.

**Methods:**

ADiGe (Abdominal Drain in Gastrectomy) Trial is a multicenter prospective randomized non-inferiority trial with a parallel design. It aimed to verify whether avoiding routine use of abdominal drain is burdened with complications, particularly an increase in postoperative invasive procedures. Patients with gastric cancer, scheduled for subtotal or total gastrectomy with curative intent, are eligible for inclusion, irrespective of previous oncological treatment.

The primary composite endpoint is reoperation or percutaneous drainage procedures within 30 postoperative days. The primary analysis will verify whether the incidence of the primary composite endpoint is higher in the experimental arm, avoiding routine drain placement, than control arm, undergoing prophylactic drain placement, in order to falsify or support the null hypothesis of inferiority. Secondary endpoints assessed for superiority are overall morbidity and mortality, Comprehensive Complications Index, incidence and time for diagnosis of anastomotic and duodenal leaks, length of hospital stay, and readmission rate.

Assuming one-sided alpha of 5%, and cumulative incidence of the primary composite endpoint of 6.4% in the control arm and 4.2% in the experimental one, 364 patients allow to achieve 80% power to detect a non-inferiority margin difference between the arm proportions of 3.6%. Considering a 10% drop-out rate, 404 patients are needed. In order to have a balanced percentage between total and subtotal gastrectomy, recruitment will end at 202 patients for each type of gastrectomy. The surgeon and the patient are blinded until the end of the operation, while postoperative course is not blinded to the patient and caregivers.

**Discussion:**

ADiGe Trial could contribute to critically re-evaluate the role of prophylactic drain in gastrectomy, a still widely used procedure.

**Trial registration:**

Prospectively registered (last updated on 29 October 2020) at ClinicalTrials.gov with the identifier NCT04227951.

**Supplementary Information:**

The online version contains supplementary material available at 10.1186/s13063-021-05102-1.

## Introduction

### Background and rationale

Prophylactic drain placement after gastrectomy has been advocated until the last few years as the main tool for early diagnosis and treatment of surgical intra-abdominal complications, especially with regard to anastomotic or duodenal stump leakages. Evidence against routine drain use after colorectal resection [1, 2] raised the interest on this argument. There was also interest for upper gastrointestinal surgery and, in 2015, a Cochrane meta-analysis [3] on 4 RCTs concluded that there was no convincing evidence to the prophylactic drain placement after gastrectomy. Nevertheless, the authors evidenced the moderate/low methodological quality of the included studies and highlighted how 3 studies out of 4 came from Eastern countries [3]. Moreover, randomization was never mentioned in 1 of the 4 articles, classified as RCTs. After 2015, other retrospective studies have been published, but most of them included a small number of patients and heterogeneous types of surgery, including multi-visceral and R2 resection [4–7]. Our study group recently published an updated meta-analysis including both RCTs and cohort studies, comparing the use of prophylactic drain with drain avoidance [8]. The results suggest that skipping drainage can reduce morbidity and length of stay, without affecting other major surgical outcomes. However, as for the Cochrane meta-analysis, the strength of this evidence is blunted by the limited quantity and quality of data available. Waiting for further evidence, the use of prophylactic abdominal drain after gastrectomy is currently left up to the surgeon’s preference.

### Objectives

The aim of the study is to evaluate whether avoiding prophylactic drain placement in gastrectomy results in an increase of postoperative invasive procedure (reoperation or percutaneous drain placement) compared with routine procedure, i.e., prophylactic drain placement. If this inferiority hypothesis was rejected, avoiding drain placement would be favored as drain placement is a possible harmful procedure. Moreover, the study will be performed on Western patients, who have been less extensively studied so far than Eastern patients.

### Trial design

The design is a multicenter non-inferiority randomized controlled trial with a parallel design, conducted on behalf of the Italian Research Group for Gastric Cancer (GIRCG). The sponsor of the study is Verona University Hospital that appointed a steering committee including members belonging to the sponsor itself and the GIRCG. The study has been approved by the Institutional Review Board (IRB) at the central coordinating center and has to be approved at each of the participating hospitals. The ADiGe trial was registered at ClinicalTrials.gov #NCT0422795. The protocol adheres to the Standard Protocol Items: Recommendations for Interventional Trials (SPIRIT) guidelines.

## Methods: Participants, interventions, and outcomes

### Study settings

All hospitals belonging to the GIRCG are eligible to participate in the ADiGe Trial, irrespective of their hospital volume. A total of 9 centers applied to participate, and, at the time of this publication, the study has already been approved by the local IRB of each center (Verona University Hospital, the leading Center, Orbassano Hospital of Turin, San Raffaele Hospital of Milan, Morgagni Hospital of Forlì, Federico II University Hospital of Naples, Niguarda Hospital of Milan, S. Orsola-Malpighi Hospital of Bologna, Parma University Hospital, Modena University Hospital).

### Eligibility criteria

All consecutive patients that undergo total (TG) or subtotal gastrectomy (STG) with curative intent, for histologically proven gastric cancer or esophago-gastric junction cancer (Siewert type II or III), are eligible in participating centers from the beginning of the study until they reach the accrual number for each arm.

#### Inclusion criteria

- Esophageal involvement ≤ 2 cm
- Patients planned for upfront surgery or treated with a neoadjuvant/perioperative chemotherapy or chemo-radiotherapy
- Open, hybrid, laparoscopic, or robotic approach
- All types of anastomoses (circular stapled, linear stapled, hand sewn)

#### Exclusion criteria

- Refuse to sign informed consent
- Age < 18
- Severe heart disease (Heart failure New York Heart Association - NYHA Class IV)
- Severe liver disease (Child-Pugh ≥ B7) [9]
- Pregnancy
- Metastatic disease
- Gastrectomy performed in an emergency condition
- Palliative surgery
- Operation different from total or subtotal oncological gastrectomy (e.g., pylorus-preserving, proximal gastrectomy)
- < D1 lymph nodal dissection according to the Japanese Gastric Cancer Association guidelines 5th edition [10]
- Reconstruction different from Roux-en-Y or Billroth II
- Multiple organ resection (except for cholecystectomy)
- Gastric cancer with duodenal involvement
- Intraoperative hyperthermic intraperitoneal chemotherapy

We included in the trial patients with limited esophageal involvement defining a threshold of 2 cm in order to guarantee a proper oncological treatment. According to the available evidences, a tumor that invades the esophagus for more than 2 cm has an increased incidence of mediastinal nodal involvement, thus requiring a thoracic approach [11, 12].

Compared to 3 RCTs already published on this topic [13–15], we also included patients that undergo neoadjuvant/perioperative therapies, as these treatments are currently the standard of care for locally advanced gastric tumors. A 2018 meta-analysis on 9 studies evidenced that neoadjuvant therapy is not associated with increased morbidity and mortality as compared to upfront surgery [16]. These results have been confirmed also in a recent propensity score matching analysis [17].

No restriction on a previous abdominal surgery has been planned since data available in literature suggest no correlation with postoperative morbidity [18, 19]. Moreover, considering that minimally invasive and open surgery outcomes are nowadays comparable, we decided to include both types of approaches.

We included only Roux-en-Y and Billroth II reconstruction because they have similar vulnerabilities: the anastomosis and the duodenal stump. On the other hand, Billroth I reconstruction has different morbidity due to the lack of a duodenal stump and a direct suture between the gastric remnant and the duodenum.

Cholecystectomy was not considered as an exclusion criterion because some evidence suggests its utility in a subset of patients undergoing gastrectomy [20], and it seems not to add morbidity or mortality to the main operation [21].

### Who will take informed consent

Patients eligible for participation are informed about the trial by one of the surgeons during the preoperative visit at the outpatient clinic. Patients can agree to participate until the day before the operation by signing the informed consent.

### Intervention description

#### Operation and intraoperative dropout

Randomized patients included in the control group (group A) receive one abdominal drain (any type of drainage is allowed) placed below the liver, passing by the duodenal stump with the apex posterior to the esophago-jejunal or gastro-jejunal anastomosis. No drainage is placed in patients assigned to the experimental group (group B). Towards the end of the operation the leading surgeon can classify a patient as an intraoperative dropout before knowing the randomization arm, if one or more of the following criteria are met:

- Intraoperative finding of non-radically resectable disease (R2 resection—gross residual disease with gross residual tumor that was not resected) [22]
- Need for unplanned multiple organ resection (except for cholecystectomy)
- High risk anastomosis defined as intraoperative test (e.g., methylene blue or pneumatic test) positive for leak or intraoperative evidence of a positive resection margin.
- Other intraoperative complication that has to be specified in the case report form (unintended intraoperative damage to major vessels and/or organs requiring reconstruction or resection, intraoperative bleeding requiring urgent transfusion, unexpected medical conditions interrupting or changing the planned procedure [23]).

#### Postoperative procedure

All patients follow the usual postoperative pathway of each participating center except for abdominal drain management. Prophylactic drain in group A patients is daily evaluated checking for suspicious debt. In patients with a normal debt, a methylene blue test (the patient is asked to drink 200 ml of water/tea with 5 ml of methylene blue) is performed on postoperative day (POD) 4 and the test is considered negative if no blue staining of drain output is apparent within 60 min (the 200-ml colored solution stays in the esophageal/gastric lumen and this is considered as an indirect sign of the absence of an anastomotic leak). After the blue test is confirmed as negative, the drain can be removed. Each center is allowed to remove drain on whatever POD after a negative test. Drain management in patients with a suspicious debt or positive blue test is left to center preference (no further blue tests are planned). Postoperative complications are recorded until POD 30 or in hospital if hospital stay is longer than 30 days.

Reoperation under general anesthesia and/or percutaneous abdominal/thoracic drain placement during the first 30 PODs for any cause related to the previous gastrectomy is considered as an event in both groups. The cause-effect relationship decision is left to the lead site surgeon and in case of doubt, the investigator can ask for support from the steering committee. This specification was considered mainly necessary in case of unrelated pathology acquired after discharge but within POD 30 (e.g., operation for road accident). After discharge, patients are evaluated in the outpatient clinic on POD 30 or in the subsequent week. Follow-up calls are conducted by authorized medical or nursing staff at POD 60 ± 7 and 90 (max + 7 days) in order to assess patient’s clinical status.

### Outcomes

The primary composite endpoint is the cumulative incidence of reoperation and/or percutaneous drainage placement by the 30th postoperative day.

Secondary endpoints are as follows:

- Incidence, severity, and time to diagnosis of anastomotic and duodenal leak
- Length of hospital stay
- Overall 30 days morbidity or in hospital if longer than 30 days
- Overall 90 days mortality
- 30 days readmission rate

Length of hospital stay is considered from the day of operation until discharge at home or at other facilities, or death. Postoperative complications, including anastomotic and duodenal leak, are classified according to the International Consensus on a Complications List After Gastrectomy for Cancer [23] and stratified by severity using the Clavien-Dindo classification [24].

### Participant timeline

**Fig. 1:**
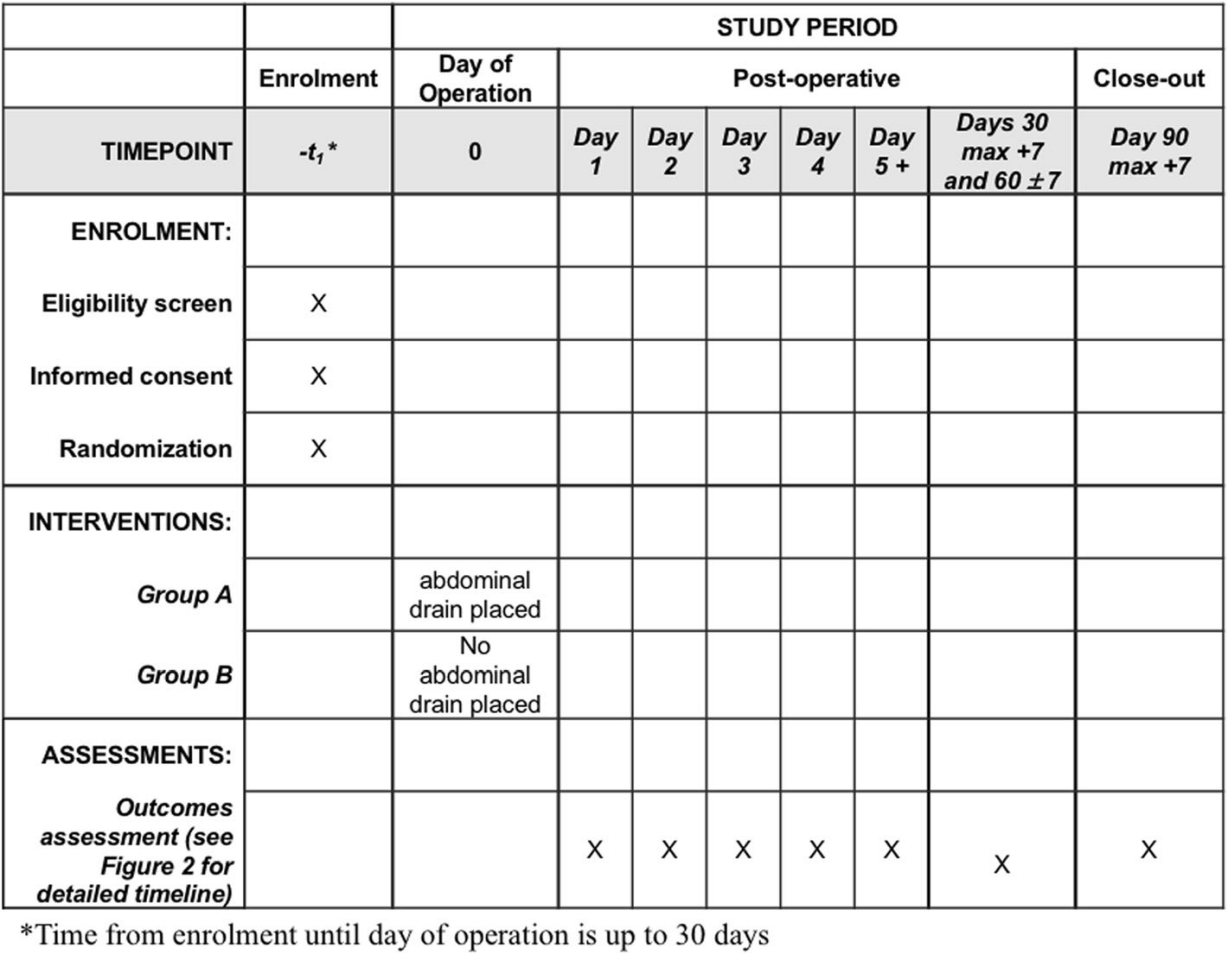
Table of assessments

**Fig. 2:**
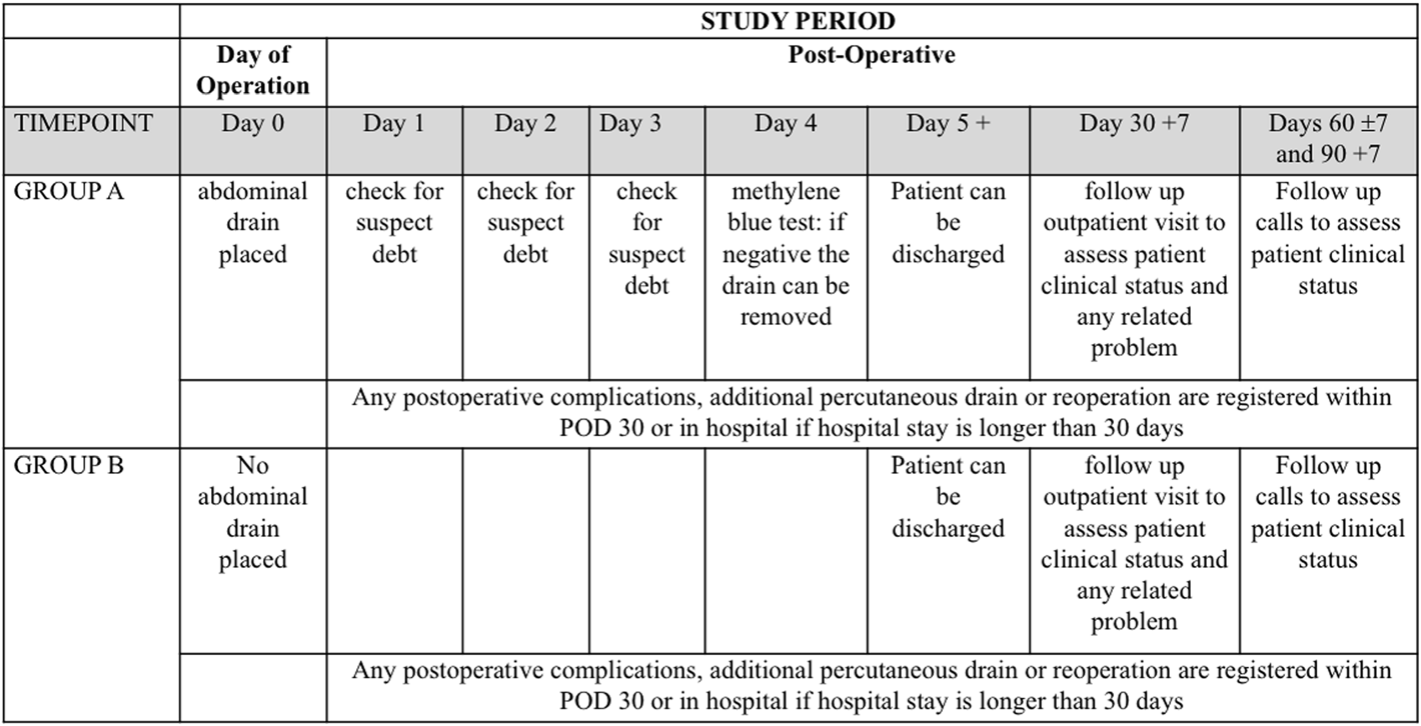
Detailed schedule of outcomes assessments

The timeline of study events is displayed in Figs. 1 and 2. After a patient’s inclusion, a medical authorized staff member randomizes the participant using an online secure module on the International Gastrectomy Complications Database website (www.gastrodata.org), the day before the operation. Upon filling out the randomization form, an immediate reply is obtained, containing the study group. “Group A” includes patients with prophylactic drain placed at the end of the operation, “group B” includes patients without any abdominal drain at the end of the operation. The ratio 1:1 is obtained using a computer-generated randomization scheme, equally stratified (1:1 ratio) for type of operation (STG or TG). Enrollment type is competitive and is capped by type of surgery.

### Sample size

According to a systematic review published in May 2020 [8], the cumulative incidence of reoperation in two RCTs was 7/115 = 6.1% (95% CI 2.5–12.1%) in the drain group and 3/115 = 2.6% (95% CI 0.5–7.4%) in the no drain group. Cumulative incidence of additional drain was 1/86 = 1.2% (95% CI 0.03–6.3%) in the drainage group and 2/84 = 2.4% (95% CI 0.3–8.3%) in the no drain group. Assuming a 70% of overlap between the two procedures in the drain group, the estimated proportion of the composite outcome is 6.44%, while assuming a 35% overlap in the no drain group, the estimated proportion of the composite outcome is 4.16%.

**Fig. 3:**
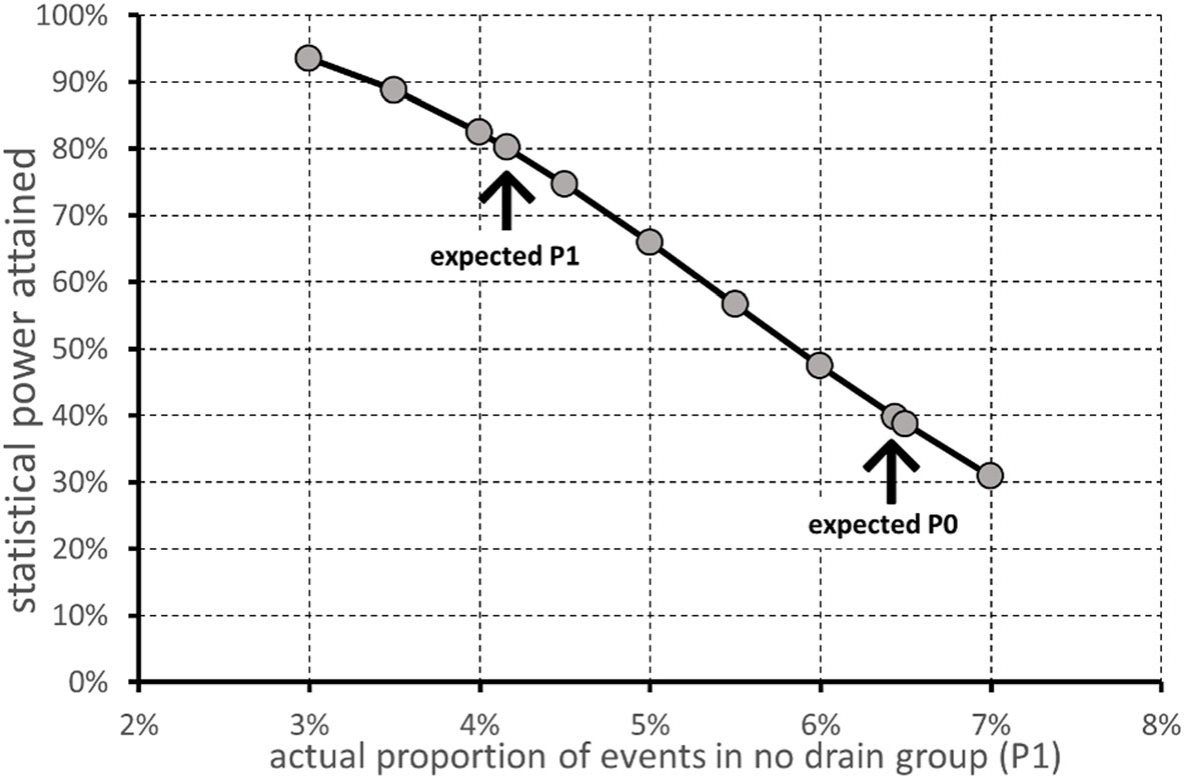
Power curve as a function of treated group proportion ranging from 3 to 7% under the alternative hypothesis of non-inferiority. P0 (proportion in the control group) = 6.4%; alpha = 0.05; n1 = n2 = 182; P1.0 (proportion in the treated group under the null hypothesis of inferiority) = 10%. The test statistic used is the one-sided *Z* test (unpooled)

Hence, we assumed a reference group proportion of 6.44%, and a treated group proportion of 10% under the null hypothesis of inferiority and 4.16% under the alternative hypothesis of non-inferiority. A sample size of 182 in each group, corresponding to 364 patients overall, achieves 80% power to detect a non-inferiority margin difference between the group proportions of 3.56% (= 10–6.44%), with a one-sided significance level of 5%. A power curve for treated group proportion ranging from 3 to 7% under the alternative hypothesis of non-inferiority is presented in Fig. 3.

Considering a 10% dropout rate, 404 patients (202 in each group) are needed. In order to have a balanced percentage between total and subtotal gastrectomy, recruitment will end at 202 patients for each type of gastrectomy.

### Assignment of interventions

#### Sequence generation and concealment mechanism

Randomization plan has been generated using www.randomization.com program, and no one of the investigators has access to the list. Participants are randomized in a 1:1 ratio, using a secure web-based randomization system to receive either intervention or routine care, stratified by type of gastrectomy.

#### Blinding

The leading surgeon and the patient are blinded to the arm assigned until gastrointestinal reconstruction is completed (and anastomosis integrity test is done, if applicable). Before discovering the arm assigned, the surgeon will decide whether the patient meet or not the dropout criteria. There is no blinding for the patient, care providers, or coordinating researcher after the operation.

### Data collection and management

All study-related information will be stored securely at each participating center. Patient’s information will be stored in locked file cabinets in areas with limited access. Digital files are kept in password-protected applications.

A section on the www.gastrodata.org secure web-based platform was developed by a specialized software firm (www.Fluxedo.com) to facilitate multicenter data collection. All data, including center, surgeon, and patient data, are strictly anonymous and managed through secure codes. Cloud servers are compliant with the General Data Protection Regulation. An SSL certificate is installed to encrypt data exchanged between applicative and users. Dataset and applicative are accessible only with personal credentials owned by authorized investigators. Each participating center has access only to its own dataset.

Data are collected until POD 91. Before recruitment process starts, investigators from each participating center will practice with the leading center data manager in order to increase proficiency in data entry and regular audits will be held with all participating centers.

Anonymization was guaranteed by removing personally direct identifiers from the dataset (e.g., using age instead of date of birth) and recoding indirect identifiers when considered at risk for a possible identification.

### Statistical methods

#### Analysis population

The primary analysis will be performed on a modified Intention-To-Treat (mITT) population, including all randomized patients who have undergone gastrectomy. Patients will be analyzed according to the treatment assigned at pre-operative randomization.

A secondary analysis will be performed on the As-Treated Population, including the same patients enclosed in the mITT population, classified according to whether they actually underwent the prophylactic drain placement or not.

Another secondary analysis will be carried out on the Per-Protocol (PP) population, including only patients undergoing surgery according to the arm assigned at randomization, and followed-up for at least 30 days. Also, patients dying before 30 days will be considered for the PP analysis.

#### Statistical analysis

Categorical variables will be presented as absolute and percent frequencies, continuous variables as mean and standard deviation when approximately symmetrically distributed, and as median and interquartile range otherwise.

The proportion of patients undergoing re-intervention or percutaneous drain placement (primary endpoint) will be computed for each group. The difference between the primary end-point proportion in the treated group and in the control group will be computed with the corresponding confidence interval. If the upper limit of the 90% confidence interval of this proportion does not exceed the non-inferiority margin difference of 3.56%, the null hypothesis of inferiority will be rejected.

Further statistical analysis on the primary endpoint will follow a hierarchical approach: if the non-inferiority null hypothesis will be rejected, significance of differences in the primary endpoint between control and experimental arms will be further investigated with a Fisher’s exact test. The relation between the primary endpoint and treatment (drain placement or avoidance) will be further investigated using a multivariable Firth’s logistic model, adjusting for center, sex, age, tumor stage, and type of gastrectomy (total/subtotal).

To evaluate significance of differences between control and experimental arms, Fisher’s exact test or chi-square test will be used for nominal variables (reoperation, placement of additional percutaneous drain, anastomotic or duodenal stump fistula, postoperative mortality, hospital readmission), Wilcoxon-Mann-Whitney test for ordinal variables (fistula or complication severity), and *t* test (or the corresponding non-parametric test) for continuous variables (length of hospital stay, time from surgery to fistula detection). Statistical significance for secondary analyses will be set at *p* < 0.05 and statistical tests will be two-sided.

Statistical analyses will be performed using Stata®/IC 16.0 for windows (StataCorp LLC, College Station, TX, USA).

### Oversight and monitoring

The sponsor in collaboration with the Steering Committee decided not to establish a formal Data Monitoring Committee for this trial considering the following 5 points:

- This trial aims to evaluate the uselessness of prophylactic drain placement after gastrectomy, a clinical practice that is still widely used but that has been already been discouraged by international guidelines
- Evidence in literature does not suggest a highly favorable or unfavorable result. The design of a non-inferiority trial was deemed necessary due to the small estimated difference between the two groups on the outcome considered for this study
- The inclusion criteria and the study design (leaving the decision on the POD of drain removal to each participating center according to their usual clinical practice) consistently reduce the (already limited) risks
- Allowing any type of drain in the study protocol (leaving the decision to each center according to their usual clinical practice) does not define this study as a device study
- Patient population is above 18 years old

All the participating centers are part of GIRCG quality of surgery and postoperative care was considered adequate for this trial according to our previous studies [25, 26].

The wide inclusion criteria and the balancing between type of resection aim to result in a trial as close as possible to the everyday Western clinical practice.

Considering that both arms include treatment commonly applied in daily clinical practice (drain placement and drain avoidance) under the inclusion criteria considered in this trial, the IRBs (of each participating center), in accordance with the legal service of all the participating center, agreed to ensure ADiGe Trial using the hospital insurance of each center. Therefore, in case of claims for damages, the responsible would be the insurance coverage of assistance activities of the center that recruited and treated the patient.

#### Safety and adverse event monitoring

Any untoward medical occurrence in a patient without regard to the possibility of a causal relationship has been defined as an adverse event (AE). Investigators from each participating center will record any AE from the day of operation until the end of the observation period. All AEs will be documented in the patient’s medical record and AE form including any workup or treatment needed and grading (according to the Common Terminology Criteria for Adverse Events), according to everyday clinical practice.

AE, expected to be related with the study treatment (bleeding from drain site, skin infection around the drain site and pain), will be also recorded in the study CRF. Any unexpected AE, considered by the investigator as causally related with the treatment, will be recorded in the study CRF and reported to the steering committee for safety reporting purposes.

AE that meets the following criteria will be recorded as serious adverse event (SAE): life-threatening condition (immediate risk of death), severe or permanent disability, and prolonged hospitalization. All SAE will be recorded in the study CRF. Moreover, the investigators will report to the local IRB and the steering committee, as soon as possible, but in no event later than 2 working days, any SAE that is deemed by the local investigator to be probably or definitely related to the study treatment. The steering committee will then review the report and send it to the promoting center’s IRB within 2 working days. SAEs, occurring after a patient is discontinued from the study, will not be reported unless the investigators will determine that they are related to the study treatment. If the number of SAE is higher than reported in the recently published Gastrodata study on complications after gastrectomy [27], patient enrollment will be terminated immediately, and the steering committee will reassess the safety of the trial.

### Ethical committee approval

The design of ADiGe Trial was approved by the scientific committee of GIRCG. The IRB of Verona has approved the present protocol with the following code: 2245CESC. Prior to implementation, any protocol amendment will be approved by GIRCG scientific committee and IRBs of each participating center. Study information in ClinicalTrials.gov will be updated accordingly.

### Dissemination policy

Results of this trial will be published in a peer-reviewed journal. The abstract will be submitted for presentations at different congresses. Within 6 months of completion of the trial, the ClinicalTrials.gov site will be updated to include summary results.

Authorship is granted to authors who make important contributions to the creation of the final publication in accordance with recommendations from the International Committee of Medical Journal Editors and GIRCG group internal policy. Authors can contribute via written or physical help in this clinical trial.

The datasets used during the current study will be available from the corresponding author on reasonable request within 1 year after completion of the trial.

**Fig. 4:**
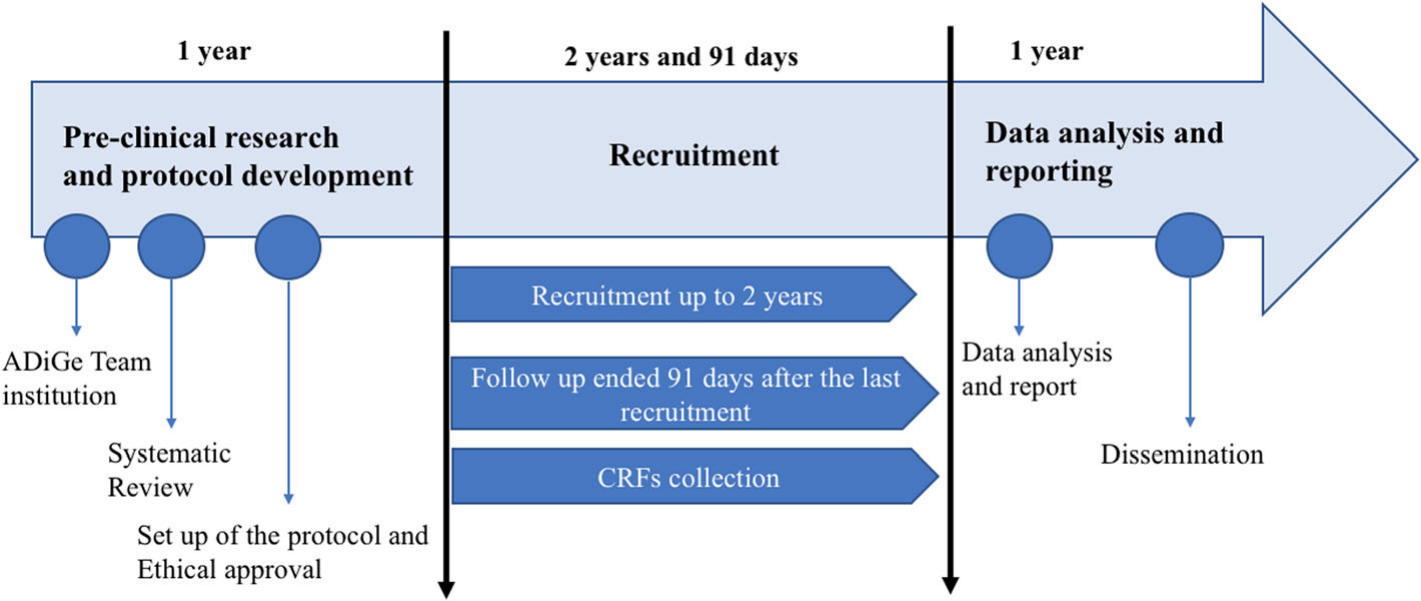
Overall timeline of the study

A timeline of the overall study design is displayed in Fig. 4.

## Discussion

Prophylactic drain in gastrectomy is still widely applied, even if some evidence against its routine use has been reported in four RCTs [13–15, 28]. Nevertheless, as highlighted in Cochrane meta-analysis, these studies lack in methodological quality and mainly come from Eastern countries [3].

So far, the only Western RCT has been published in 2005 by Alvarez and included 60 patients that underwent total gastrectomy [13]. The author reported a significantly longer hospital stay (18.8 vs 12.9 days) and higher morbidity rate (37.9% vs 9.7%) in the drain group. Reoperation rate was rather high in both groups, with a trend in favor of no drain population (9.7% vs 24.1%). However, the results of this study are jeopardized by the small sample size, unclear primary outcome, and inclusion of multivisceral resection.

The largest Eastern RCT has been published by Jiang and included a total of 170 patients treated with total or subtotal gastrectomies aimed to compare complication rate between the drain and no drain groups [14]. The trial resulted in a comparable morbidity rate with a total of three patients with intra-abdominal abscess (one in the drain, two in the no drain group) that required ultrasound-guided percutaneous drainage.

A multi-institutional analysis from US on 344 patients that underwent total gastrectomy observed no differences in overall complication rate and 30 day mortality. Need for secondary drain placement (10% vs 9%) or reoperation (13% vs 8%) were also comparable [6].

Based on these limited evidence, we designed the ADiGe Trial aiming to contribute to a critical re-evaluation of the role of prophylactic drain placement in gastrectomy. Of note, the ADiGe Trial is the first RCT studying the use of prophylactic drain in gastrectomy in a large cohort of Western patients.

### Trial status

Protocol Version: 2 (20 October 2019)

Recruitment began: December 23, 2019

Approximate date of recruitment completion: December 31, 2021

## Supplementary Information

**Additional file 1.**

## Data Availability

The full data set will be hosted in the www.gastrodata.org site. All principal investigators will be given access to their own site’s data sets; access to other sites’ data upon request only will be granted only after the completion of the recruitment phase. Data will be coded so that anonymity is preserved. The data will be kept in storage for 15 years.

## Abbreviations

STG: Subtotal gastrectomy
TG: Total gastrectomy
ADiGe: Abdominal Drain in Gastrectomy
POD: Postoperative day
RCT: Randomized controlled trial
