# Supplementary material for: Utility of Abdominal Drain in Gastrectomy (ADiGe) Trial: study protocol for a multicenter non-inferiority randomized trial
Source: Trials. 2021 Feb 17;22:152. doi: 10.1186/s13063-021-05102-1 (PMC7891135; doi:10.1186/s13063-021-05102-1)
Supplement: Supplementary file 1 — Additional file 1. [file 13063_2021_5102_MOESM1_ESM.docx]

Verona, December the 2^nd^ 2020

**ANALYSES PERFORMED WITH THE SOFTWARE STATA, ONLY ON RCT DATA**

REOPERATION IN “DRAIN GROUP”

. cii prop 115 7

-- Binomial Exact --

Variable | Obs Proportion Std. Err. [95% Conf. Interval]

-------------+---------------------------------------------------------

| 115 .0608696 .0222953 .0248204 .1213973

REOPERATION IN “NO DRAIN GROUP”

. cii prop 115 3

-- Binomial Exact --

Variable | Obs Proportion Std. Err. [95% Conf. Interval]

-------------+---------------------------------------------------------

| 115 .026087 .0148636 .0054124 .0743462

ADDITIONAL DRAIN IN “DRAIN” GROUP

. cii prop 86 1

-- Binomial Exact --

Variable | Obs Proportion Std. Err. [95% Conf. Interval]

-------------+---------------------------------------------------------

| 86 .0116279 .0115601 .0002943 .0630905

ADDITIONAL DRAIN IN “NO DRAIN” GROUP

. cii prop 84 2

-- Binomial Exact --

Variable | Obs Proportion Std. Err. [95% Conf. Interval]

-------------+---------------------------------------------------------

| 84 .0238095 .0166342 .0028966 .0833745

**ANALYSES PERFORMED WITH THE SOFTWARE PASS, ONLY ON RCT DATA**

Hintze J (2004). NCSS and PASS. Number Cruncher Statistical Systems. Kaysville, Utah. [www.ncss.com](http://www.ncss.com)

**Power Analysis of Non-Inferiority Tests of Two Independent Proportions**

Page/Date/Time 1 10/12/2020 13:10:39

**Numeric Results for Non-Inferiority Tests Based on the Difference: P1 - P2**

**H0: P1-P2>=D0. H1: P1-P2=D1<D0. Test Statistic: Z test (unpooled)**

**Sample Sample Equiv. Actual Equiv. Actual**

**Size Size Grp 2 Grp 1 Grp 1 Margin Margin**

**Grp 1 Grp 2 Prop Prop Prop Diff Diff Target Actual**

**Power N1 N2 P2 P1.0 P1.1 D0 D1 Alpha Alpha Beta**

0,9351 182 182 0,0644 0,1000 0,0300 0,0356 -0,0344 0,0500 0,0649

0,8879 182 182 0,0644 0,1000 0,0350 0,0356 -0,0294 0,0500 0,1121

0,8245 182 182 0,0644 0,1000 0,0400 0,0356 -0,0244 0,0500 0,1755

0,8017 182 182 0,0644 0,1000 0,0416 0,0356 -0,0228 0,0500 0,1983

0,7470 182 182 0,0644 0,1000 0,0450 0,0356 -0,0194 0,0500 0,2530

0,6592 182 182 0,0644 0,1000 0,0500 0,0356 -0,0144 0,0500 0,3408

0,5665 182 182 0,0644 0,1000 0,0550 0,0356 -0,0094 0,0500 0,4335

0,4742 182 182 0,0644 0,1000 0,0600 0,0356 -0,0044 0,0500 0,5258

0,3977 182 182 0,0644 0,1000 0,0644 0,0356 0,0000 0,0500 0,6023

0,3869 182 182 0,0644 0,1000 0,0650 0,0356 0,0006 0,0500 0,6131

0,3080 182 182 0,0644 0,1000 0,0700 0,0356 0,0056 0,0500 0,6920

Note: exact results based on the binomial were only calculated when both N1 and N2 were less than 100.

**References**

Chow, S.C.; Shao, J.; Wang, H. 2003. Sample Size Calculations in Clinical Research. Marcel Dekker. New York.

Farrington, C. P. and Manning, G. 1990. 'Test Statistics and Sample Size Formulae for Comparative Binomial Trials with Null Hypothesis of Non-Zero Risk Difference or Non-Unity Relative Risk.' Statistics in Medicine, Vol. 9, pages 1447-1454.

Fleiss, J. L., Levin, B., Paik, M.C. 2003. Statistical Methods for Rates and Proportions. Third Edition. John Wiley & Sons. New York.

Gart, John J. and Nam, Jun-mo. 1988. 'Approximate Interval Estimation of the Ratio in Binomial Parameters: A Review and Corrections for Skewness.' Biometrics, Volume 44, Issue 2, 323-338.

Gart, John J. and Nam, Jun-mo. 1990. 'Approximate Interval Estimation of the Difference in Binomial Parameters: Correction for Skewness and Extension to Multiple Tables.' Biometrics, Volume 46, Issue 3, 637-643.

Lachin, John M. 2000. Biostatistical Methods. John Wiley & Sons. New York.

Machin, D., Campbell, M., Fayers, P., and Pinol, A. 1997. Sample Size Tables for Clinical Studies, 2^nd^ Edition. Blackwell Science. Malden, Mass.

Miettinen, O.S. and Nurminen, M. 1985. 'Comparative analysis of two rates.' Statistics in Medicine 4: 213-216.

**Report Definitions**

'Power' is the probability of rejecting a false null hypothesis. It should be close to one.

'N1 and N2' are the sizes of the samples drawn from the corresponding groups.

'P2' is the response rate for group two which is the standard, reference, baseline, or control group.

'P1.0' is the smallest treatment-group response rate that still yields a non-inferiority conclusion.

'P1.1' is the treatment-group response rate at which the power is calculated.

'Target Alpha' is the probability of rejecting a true null hypothesis that was desired.

'Actual Alpha' is the value of alpha that is actually achieved.

'Beta' is the probability of accepting a false H0. Beta = 1 - Power.

'Grp 1' refers to Group 1 which is the treatment or experimental group.

'Grp 2' refers to Group 2 which is the reference, standard, or control group.

'Equiv.' refers to a small amount that is not of practical importance.

'Actual' refers to the true value at which the power is computed.

**Summary Statements**

Sample sizes of 182 in group one and 182 in group two achieve 80% power to detect a non-inferiority margin difference between the group proportions of 0,0356. The reference group proportion is 0,0644. The treatment group proportion is assumed to be 0,1000 under the null hypothesis of inferiority. The power was computed for the case when the actual treatment group proportion is 0,0416. The test statistic used is the one-sided Z test (unpooled). The significance level of the test was targeted at 0,0500. The significance level actually achieved by this design is NA.

**Chart Section**
